# Continuing education for systematic reviews: a prospective longitudinal assessment of a workshop for librarians

**DOI:** 10.5195/jmla.2020.492

**Published:** 2020-01-01

**Authors:** Barbara L. Folb, Mary L. Klem, Ada O. Youk, Julia J. Dahm, Meiqi He, Andrea M. Ketchum, Charles B. Wessel, Linda M. Hartman

**Affiliations:** Librarian Emeritus, Health Sciences Library System, University of Pittsburgh, Pittsburgh, PA, folb@pitt.edu; Research and Instruction Librarian and Liaison to the School of Nursing, Health Sciences Library System, University of Pittsburgh, Pittsburgh, PA, klem@pitt.edu; Associate Professor, Graduate School of Public Health, Department of Biostatistics, University of Pittsburgh, Pittsburgh, PA, ayouk@pitt.edu; Coordinator of Technology Integration Services, Health Sciences Library System, University of Pittsburgh, Pittsburgh, PA, jdahm@pitt.edu; Data Analyst, Department of Pharmacy and Therapeutics, University of Pittsburgh, Pittsburgh, PA, meh206@pitt.edu; Research and Instruction Librarian and Scholarly Communication Liaison, Health Sciences Library System, University of Pittsburgh, Pittsburgh, PA, ketchum@pitt.edu; Head of Research Initiative, Health Sciences Library System, University of Pittsburgh, Pittsburgh, PA, cbw@pitt.edu; Glenshaw, PA, linda@hartman-team.com

## Abstract

**Objective:**

This prospective, longitudinal study explored the impact of a continuing education class on librarians’ knowledge levels about and professional involvement with systematic reviews. Barriers to systematic review participation and the presence of formal systematic review services in libraries were also measured.

**Methods:**

Participants completed web-based surveys at three points in time: pre-class, post-class, and six-months’ follow-up. Descriptive statistics were calculated for demographics and survey questions. Linear mixed effects models assessed knowledge score changes over time.

**Results:**

Of 160 class attendees, 140 (88%) completed the pre-class survey. Of those 140, 123 (88%) completed the post-class survey, and 103 (74%) completed the follow-up survey. There was a significant increase (*p*<0.00001) from pre-class to post-class in knowledge test scores, and this increase was maintained at follow-up. At post-class, 69% or more of participants intended to promote peer review of searches, seek peer review of their searches, search for grey literature, read or follow published guidelines on conduct and documentation of systematic reviews, and ask for authorship on a systematic review. Among librarians who completed a systematic review between post-class and follow-up, 73% consulted published guidelines, 52% searched grey literature, 48% sought peer review, 57% asked for authorship, and 70% received authorship.

**Conclusions:**

Attendance at this continuing education class was associated with positive changes in knowledge about systematic reviews and in librarians’ systematic review–related professional practices. This suggests that in-depth professional development classes can help librarians develop skills that are needed to meet library patrons’ changing service needs.

## INTRODUCTION

Continuing education (CE) courses can be a vital resource for staying up to date in environments that are undergoing rapid scientific, technological, or financial changes. CE activities are used extensively in the health sciences, and evaluations of the impact of CE training on various health professionals’ knowledge levels and clinical practice have been described in detail [1].

However, less is known about the impact of CE on medical librarians. Much of the literature published to date has primarily focused on assessing librarians’ satisfaction with and preferences for CE activities [2–4]. Satisfaction surveys are useful for describing participants’ general reactions to CE training and for eliciting suggestions for improving the training experience. To determine the extent to which learning has occurred, however, an evaluation should include assessment of changes in knowledge levels and on-the-job behaviors [5]. To the best of the authors’ knowledge, there are no studies that have prospectively evaluated these outcomes for librarians attending a CE course or workshop.

Systematic reviews are an increasingly prevalent type of research project [6] and represent an opportunity for medical or health sciences libraries to promote and expand the services that they offer to their communities [7]. Cooper and Crum analyzed reports of health sciences librarians’ traditional and new activities and identified “systematic review librarian” as an emerging role for this group [8]. In a survey study of biomedical libraries, Crum and Cooper found that 58% of library directors reported having only recently started to offer support services for systematic reviews, while 22% were planning to support systematic reviews in the future [9]. A significant percentage of directors also reported that “lack of knowledge or skills” (31%) and “lack of time for education or training” (29%) were barriers for staff librarians faced with performing systematic reviews and other new and emerging activities.

A team of faculty librarians at the University of Pittsburgh Health Sciences Library System (HSLS) developed an internal training program to enhance colleagues’ ability to collaborate on systematic review projects and to complete comprehensive, high-quality literature searches. Beginning in 2009, this training was offered to the larger librarian community as a 2.5-day CE class (“Systematic Review Workshop: The Nuts and Bolts for Librarians”). This face-to-face class, which was offered from 2009 to 2019, was highly interactive and utilized a blend of lectures, small group activities, and extensive group discussions. Participants received all class materials on a universal serial bus (USB) drive, including PowerPoint files and uniform resource locator (URL) links and references cited during the class. Librarians who completed the class received 20 CE credits from the Medical Library Association. As of November 2017, over 600 librarians from the United States, Canada, and other countries have attended the class.

A retrospective evaluation of early attendees (individuals who attended the HSLS class between November 2009 and April 2012; n=169) of the HSLS class suggested that participation had a positive impact on participants’ professional practice [10]. Nearly 72% reported working on at least 1 systematic review after participating in the class, 71% felt confident that they could complete a high-quality systematic review, and 42% reported positive changes in their libraries’ support of systematic reviews. Given the retrospective nature of the study, however, it was still unclear to what extent class participation influenced librarians’ understanding of, knowledge about, and on-the-job involvement with systematic review projects. Thus, the purpose of this study was to prospectively evaluate the impact of the HSLS systematic review class on the knowledge and professional practices of participants who attended the workshop after April 2012.

## METHODS

### Study participants

Longitudinal survey respondents were 140 of the 160 librarians and information specialists who enrolled in the HSLS systematic review class between July 2012 and April 2014.

### Survey design

The previously reported retrospective survey [10] and the prospective surveys were created simultaneously and contained many of the same questions, allowing the retrospective study analysis to inform analysis of the prospective study. Survey design was led by a librarian with formal survey design training. All workshop instructors contributed ideas on what to measure and contributed to discussion on what types of questions to use. Longitudinal prospective evaluations of CE courses in other disciplines provided some examples of logical question structure, but no questions were copied from other surveys.

The prospective longitudinal study was designed to measure retention of knowledge and changes in professional practices over six months using three surveys. The pre-class survey measured participants’ pre-class knowledge, confidence, behavior, motivation, demographics, and institutional characteristics (supplemental Appendix A). The post-class survey measured their future intentions to participate in systematic reviews, use selected systematic review practices, and seek further education, as well as changes in their confidence and knowledge (supplemental Appendix B). The follow-up survey measured changes in their confidence, knowledge, and follow-through on post-class intentions six months after the class (supplemental Appendix C). A summary of relationships between questions in the three surveys is available in Table 1 in supplemental Appendix D. This study was deemed exempt from review by the University of Pittsburgh Institutional Review Board.

The surveys assessed respondents’ professional systematic review practices and knowledge of the systematic review process before and after the workshop. Examples of systematic review practices that were measured were grey literature searching, peer review of search strategies, use of published guidelines for documenting the review’s search strategies (e.g., Preferred Reporting Items for Systematic Reviews [PRISMA] [11]), requests for authorship, and familiarity with *Finding What Works in Health Care: Standards for Systematic Reviews*, a well-known Institute of Medicine (IOM) report on designing, conducting, and reporting systematic reviews [12].

To assess participants’ knowledge, workshop instructors identified the most important content from their sections of the workshop and proposed questions pertaining to that content, resulting in 11 questions about specific aspects of literature searches for systematic reviews with true/false, multiple choice/one response, or multiple choice/choose all that apply response formats. Use of multiple-choice questions, with the instruction to “check all that apply,” resulted in 19 possible answers that could be graded. Knowledge questions received 1 point for each correct response, with overall knowledge score reflected as a percentage (# of correct responses/19).

Survey validation was limited to face validity testing. Survey questions were tested on paper by two groups of HSLS librarians: those who taught the workshop and those who did not. Surveys were modified as needed for clarity, question flow, and branching logic. LimeSurvey, an open source survey application, was used to create the surveys and manage online survey administration [13].

### Survey administration

Approximately two weeks prior to attending the class, all participants who had enrolled in the workshop sessions between July 2012 and April 2014 received an email invitation to participate in a survey study of the impact of class training. Individuals who agreed to participate in the study used a link in the invitation to access an initial survey (pre-class) and were subsequently sent emails containing survey links immediately at the end of the class (post-class) and six months after the end of the class (follow-up). The post-class and follow-up surveys were available to participants for two weeks, and up to two reminder emails were sent to nonresponders. Any participant who failed to answer all items on the pre-class or post-class survey or who declined further participation at either point received no further emails or surveys.

### Statistical analysis

Survey responses were exported from LimeSurvey as a comma delimited file and imported into Excel for data cleaning. Cleaned data were imported into STATA, version 14, for analysis [14]. Participation rates were calculated across the survey time points (pre-class, post-class, follow-up), and analyses were performed to assess whether baseline variables were related to survey completion status, using Fisher’s exact test if there were fewer than five responses in a cell or chi-squared tests if there were more than five. Descriptive statistics were computed for all demographic and baseline (pre-class) variables, using frequencies and percentages for categorical variables and means and standard deviations for the continuous knowledge scores. Open-ended text questions were analyzed qualitatively, and themes were identified. Tests of proportions using Z-tests were performed to assess changes in the proportions of positive responses between the pre-class and follow-up surveys. Linear mixed effects models using chi-squared tests were used to assess whether knowledge increased over time using knowledge score as the outcome and time as a categorical main effect.

## RESULTS

### Response rate

Of the 160 individuals who attended the HSLS systematic review class between July 2012 and April 2014, 140 (88%) responded to the email invitation to participate in the study and submitted a usable pre-class survey. Of those 140 individuals, 123 (88%) completed the post-class survey, and 103 (84%) of 122 invited participants completed the follow-up survey. The overall response rate pre-class to follow-up was 64% (103/160) (Table 2 in supplemental Appendix D). Participants who completed the pre-class survey but did not complete the post-class or follow-up survey (nonresponders) were compared to participants who completed all 3 surveys (responders). No statistically significant differences were found between responders and nonresponders on any professional or institutional characteristics (*p*>0.05).

### Professional and institutional characteristics

Most (79%) respondents to the pre-class survey worked in user services or reference positions, 9% were in management positions, and 12% reported a variety of other job types (Table 3 in supplemental Appendix D). Over half (58%) of respondents to the pre-class survey had been in the library profession for 10 years or less. Most (94%) had a master’s degree in library science, 24% had a second subject masters, and 4% had a doctorate (PhD) or doctoral-level professional degree (Table 3 in supplemental Appendix D). Most respondents (67%) worked in academic health sciences libraries, whereas 11% worked in hospital libraries, 11% in non–health sciences academic libraries, 6% in government libraries, and 5% in other work settings such as research organizations (Table 4 in supplemental Appendix D).

### Motivation for workshop attendance

Seven primary themes were identified in the 133 free-text answers to the pre-class question, “What is the most important reason you are here today?” From most to least frequently mentioned, they were increase personal systematic review skills (n=86), increase personal knowledge about systematic reviews (n=63), apply increased knowledge to collaboration with and teaching patrons (n=61), use class knowledge to plan or improve library services (n=32), gain confidence in their own systematic review skills (n=13), apply class content for library staff development (n=11), and be a principle investigator or author on systematic reviews (n=4). The most frequently mentioned specific skill that respondents wanted to improve was literature searching (n=31).

### Confidence

At pre-class, only 19% of respondents reported feeling confident that they could complete a high-quality systematic review, and 46% felt confident they could effectively explain the subtleties and nuances of literature searching to library users. At post-test, 66% of respondents reported feeling confident about completing a high-quality systematic review, and 96% were confident they could communicate effectively with library users. This increase in confidence appeared to be maintained at follow-up: 70% were confident in their ability to complete a high-quality review, and 95% were confident they could communicate effectively with library users (Table 1).

**Table 1:** Confidence in systematic review skills

	Pre-class(n=140)		Post-class(n=123)		Follow-up(n=103)	

	n	(%)	n	(%)	n	(%)
Prompt: I can complete a high-quality systematic review search.						
Strongly agree/agree	26	(19%)	81	(66%)	72	(70%)
Neutral	51	(36%)	35	(28%)	27	(26%)
Disagree/Strongly disagree	62	(44%)	6	(5%)	4	(4%)
Missing	1	(<1%)	1	(<1%)	0	—
Prompt: As a consultant, I can communicate to library users the nuances and subtleties of systematic review searching.						
Strongly agree/agree	64	(46%)	118	(96%)	98	(95%)
Neutral	35	(25%)	4	(3%)	5	(5%)
Disagree/Strongly disagree	27	(19%)	1	(<1%)	0	—
Don’t know	8	(6%)	0	—	0	—
Missing	6	(4%)	0	—	0	—

Post-class systematic review work did not influence respondents’ confidence in communicating systematic review information at any measurement point (Fisher’s exact tests, pre-class: *p=*0.106, post-class: *p=*0.739, follow-up: *p=*0.878). However, post-class systematic review work was associated with significantly higher confidence in completing a high-quality systematic review at post-class and follow-up (Fisher’s exact tests, pre-class: *p=*0.122, post-class: *p=*0.046, follow-up: *p=*0.001).

### Knowledge

Mean knowledge scores for all respondents significantly increased over time, from 70% at pre-class to 85% at post-class and 84% at follow-up (pre-class to post-class, χ^2^(1)=152.39, *p*<0.0001; pre-class to follow-up, χ^2^(2)=179.56, *p*<0.00001; post-class to follow-up, χ^2^(1)=1.61, *p*=0.204; Table 2), reflecting overall positive changes in respondents’ knowledge about database choices, search filters, use of grey literature, and other aspects of systematic review methodology as well as high knowledge retention over time. The pattern of knowledge score changes from pre-class to follow-up was the same for those who worked on a systematic review after class and those who did not (χ^2^(2)=2.80, *p=*0.247), suggesting that other factors besides reported systematic review work supported knowledge retention.

**Table 2:** Comparison of total knowledge scores over time

Measure	Pre-class(n=137)	(SD)	Post-class(n=123)	(SD)	Follow-up(n=101)	(SD)
Range	6–18		7–19		7–19	
Mean	13.37	(2.7)	16.21	(2.4)	15.96	(2.2)
# of correct answers/19	70%		85%		84%	

### Behaviors

**Figure 1:**
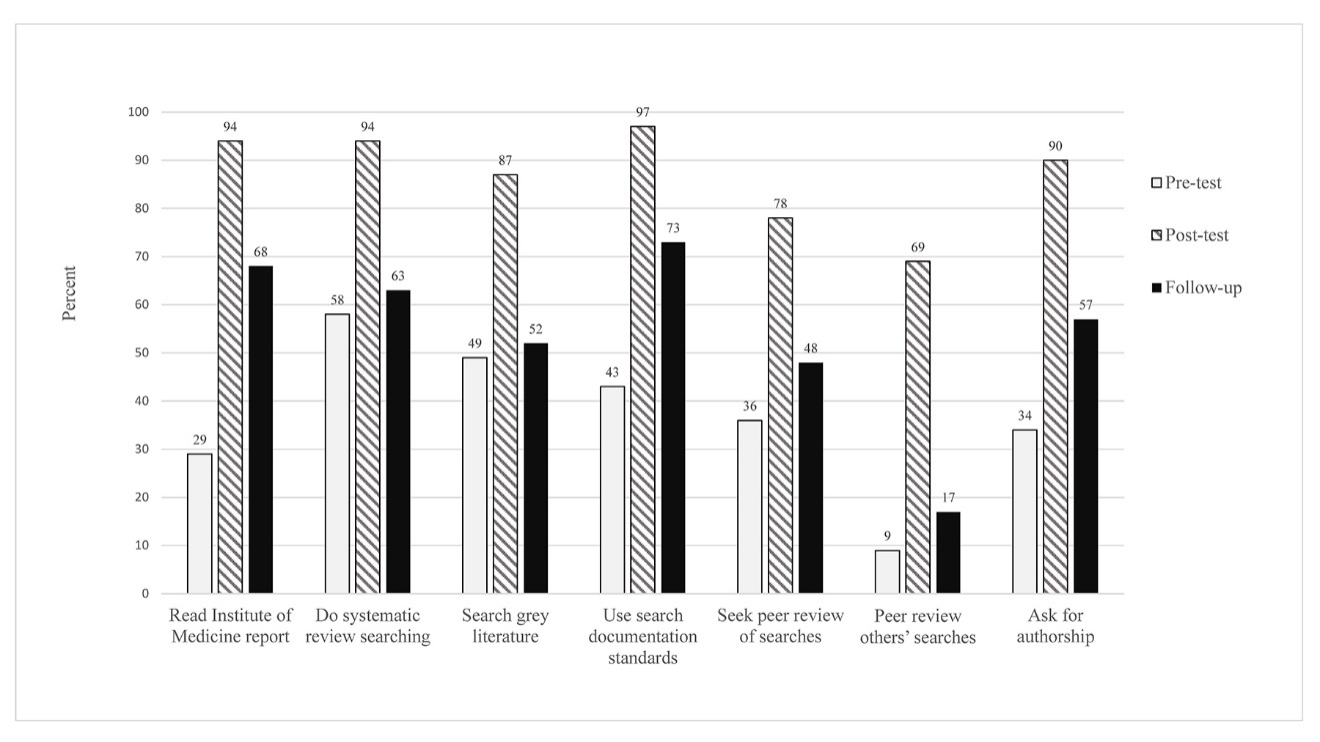
Systematic review behaviors (reported at pre-class and follow-up) and intention to engage in behaviors (reported at post-class)

At pre-class, relatively few respondents had read part or all of the IOM reports (29%) or used published guidelines such as PRISMA to document their search strategies (43%). At post-class, respondents’ interest in engaging in these review-related behaviors was very high: 94% intended to read the IOM report (94%), and 97% intended to use PRISMA documentation guidelines (Figure 1). At follow-up, a significantly greater proportion (68%) of all respondents had read all or part of the IOM report (Z=−5.93, *p<*0.0001) and were more likely to report using documentation guidelines (*t*=5.16, *p*<0.0001; Table 5 in supplemental Appendix D).

A somewhat different pattern was observed for peer review of search strategies. At pre-class, only 9% of respondents had ever provided peer review for other librarians, and 36% had sought review of their own strategies. At post-class, more than three-quarters (78%) intended to seek peer review of their own searches, and 69% intended to promote the use of peer review of searches to other librarians (Figure 1). However, at follow-up, among respondents who had worked on a systematic review since attending the class, there were no significant increases in the proportions of those seeking review of their search strategies (48%; Z=−1.39, *p=*0.164) or providing peer review of another librarian’s search (17%; Z=−1.66, *p=*0.098; Table 5 in supplemental Appendix D).

Similar results were observed for use of grey literature searches. At pre-class, among respondents who had previous experience working on a systematic review (n=79), nearly half (49%) had completed grey literature searches, and their post-class intention to conduct such searches was high (87%). However, at follow-up, among respondents who had worked on a systematic review, only 52% reported including grey literature searching (Z=−0.45, *p=*0.655).

Overall, the proportion of respondents asking for authorship increased from 34% (27/80) at pre-class to 57% (37/65) at follow-up. Among the 65 respondents who conducted a systematic review after attending the class, there was a statistically significant difference (χ^2^(1)=7.3, *p*=0.025) in the proportion asking for authorship at follow-up across 3 categories: those who conducted systematic reviews before class and asked for authorship (14/17, 82%), those who conducted systematic reviews before class and did not ask for authorship (12/29, 41%), and those who participated in their first systematic reviews after the class (11/19, 58%). When the 26 who reported conducting a systematic review both before and after the class were collapsed into a single group for analysis, there was no significant difference in the proportion asking for authorship at follow-up among these individuals (26/46, 57%) and those who had conducted their first systematic review after the class (11/19, 58%). Sample sizes were not large enough to identify librarian characteristics correlated with asking for authorship.

### Educational efforts

At pre-class, 37% of respondents had attended any systematic review-related trainings, and 16% had taken for-credit classes in subject areas that were relevant to systematic reviews, such as statistics, epidemiology, research methods, evidence-based health, or health literature appraisal. At post-class, 66% of respondents intended to attend more systematic review trainings in the future, 100% agreed that they would use HSLS class materials after class, and 94% intended to share the materials with their colleagues. At follow-up, 29% had attended other systematic review trainings, and 10% had enrolled in for-credit classes relevant to systematic reviews. Changes in participation in trainings and for-credit classes from pre-class to follow-up were not statistically significant (*p*>0.05; Table 6 in supplemental Appendix D). Most (83%) respondents shared HSLS class materials with their colleagues (Table 6 in supplemental Appendix D).

The percentage of respondents who reported engaging in educational activities on systematic review topics with their colleagues at home fell from 61% pre-class to 43% at follow-up (43%). Among the respondents who reported engaging in these activities (pre-class, n=86; follow-up, n=44), the most popular were attending local workshops (pre-class: 51%; follow-up: 27%), one-on-one mentoring (pre-class: 40%; follow-up: 50%), and journal club (pre-class: 24%; follow-up: 23%) (Table 7 in supplemental Appendix D).

### Institutional changes

At pre-class, 29% of respondents’ libraries had a formal systematic review service in place for 4 years on average. Of the 71% of respondents whose libraries did not have a formal systematic review service, 48% had plans to implement one. Regardless of whether their systematic review services were formalized, only 43% of respondents’ libraries actively promoted the ability of their librarians to assist with systematic reviews. Out of that 43%, 67% reported promoting the services on the library websites, with lower percentages of respondents’ libraries employing written communications (22%), presentations (15%), and librarian interactions with patrons (10%).

At follow-up, 31% of respondents had made a change in the systematic review services offered at their institutions since attending the class. Of those making a change, 62% added a systematic review service, and the remaining 38% made organizational or administrative changes to their existing services, such as personnel changes or additions, website revisions, or service evaluations (Table 8 in supplemental Appendix D). Systematic review services were promoted by 50% of respondents, with website remaining the most frequent form of promotion (71%). Among respondents reporting a change in their institutional service, 88% believed that the class contributed to the reported changes to some or a great degree.

### Barriers to participation in systematic reviews

At pre-class, the most frequently reported barrier to participation in a systematic review was lack of knowledge (66%), followed by researchers’ lack of knowledge about systematic review methodology (64%), and researchers’ failure to ask for librarian involvement in systematic reviews (51%). At follow-up, only 9% of respondents cited lack of knowledge as a barrier, which was a significant decline (Table 3). However, researchers’ lack of knowledge about systematic review methodology (59%) and failure to ask for librarian involvement in systematic reviews (55%) remained the most frequently reported barriers.

**Table 3:** What barriers, if any, have you encountered to being involved in systematic review searching?

Barriers (multiple answers possible)	Pre-class(n=140)			Follow-up(n=103)			Change from pre-class to follow-up	

	n	(%)	Rank	n	(%)	Rank	Z-test	*p* value
Knowledge	93	(66%)	1	9	(9%)	6	Z=9.00	*p*<0.0001
Users don’t understand	89	(64%)	2	61	(59%)	1	Z=0.68	*p*=0.491
Users don’t ask	72	(51%)	3	57	(55%)	2	Z=−0.60	*p=*0.546
Time	66	(47%)	4	54	(52%)	3	Z=−0.66	*p=*0.506
Low staff	39	(28%)	5	44	(43%)	4	Z=−2.41	*p=*0.016
Other duties	31	(22%)	6	28	(27%)	5	Z=−0.73	*p=*0.462
Too many requests	12	(9%)	7	9	(9%)	6	Z=−0.04	*p=*0.964
Administrative support	7	(5%)	8	9	(9%)	6	Z=−1.16	*p=*0.246
None	4	(3%)	9	3	(3%)	7	Z=−0.03	*p=*0.980
Don’t know	4	(3%)	9	1	(<1%)	9	—	
Missing database*	1	(<1%)	10	2	(2%)	8	—	
Administration doesn’t understand*	1	(<1%)	10	1	(<1%)	9	—	
Library changes for services*	0	—	—	1	(<1%)	9	—	
Researchers don’t complete project*	0	—	—	1	(<1%)	9	—	
Emotion-stress, overwhelmed*	0	—	—	1	(<1%)	9	—	

* Derived from free text responses to list other barriers.

## DISCUSSION

### Retention of knowledge and confidence

Individuals attending the HSLS CE class on systematic reviews were motivated to do so by a desire to increase their knowledge about systematic reviews, their skills in performing systematic reviews, and their ability to communicate with library patrons about this research methodology. The results of our study suggest that attendance at the class was associated with positive changes in the medical librarians’ knowledge levels. Respondents’ mean knowledge scores immediately after the end of class were significantly higher than their pre-class scores, and this increase in knowledge was maintained over a six-month period of time, regardless of whether the respondents worked on a systematic review project during that period of time. This positive change was also reflected in reports of barriers to participation in systematic reviews: lack of knowledge was most frequently reported in the pre-class survey but at follow-up was reported by fewer than 10% of respondents.

Attendance at the class was associated with positive changes in respondents’ confidence levels. All respondents reported increases in confidence about communicating with researchers, and this increase remained steady over time, regardless of librarians’ experience with systematic review projects after the end of class. Maintaining confidence in untested communication skills might sound illogical, but it is possible that several factors contributed. First, librarians could be teaching about systematic reviews even if they are not doing them. Second, they have materials from the class that they could consult to refresh their knowledge should they be asked about the topic. This suggests that even librarians who are in settings with limited demand for assistance with systematic reviews can benefit from training and be more prepared to respond to future requests from patrons.

Confidence in their ability to complete high-quality systematic reviews also rose among respondents and was significantly associated with post-class work on a systematic review. Librarians who had not tested their systematic review skills in the real world were more likely to be neutral about their confidence. This suggests that while the workshop may immediately enhance participants’ knowledge about systematic reviews, actual hands-on involvement may be required to increase or maintain confidence in their ability to apply this new knowledge in practice over time.

### Behavioral change

Measuring behavioral change over time, an important indicator of training impact, was a high priority of our study. Perhaps not surprisingly, post-class intention to engage in professional behaviors was high, suggesting that the workshop effectively presented the importance of these behaviors. Due to the uniformly high levels of intentions observed at post-class, we were not able to examine the impact of intention levels on respondents’ reported behaviors at follow-up.

We were able, however, to examine follow-through on post-class intentions among respondents who worked on a systematic review after attending the class. Working on a review had no effect on intention to read the IOM report or use documentation guidelines: all respondents were more likely to have engaged in these behaviors at follow-up than at pre-class. Librarians who had worked on a review after attending the class were no more likely to have sought peer review, to have conducted peer review for another librarian, or to have searched grey literature. This lack of effect might reflect the amount of limited control a librarian has on decisions to use these strategies. For example, a librarian may struggle to find a colleague willing to conduct peer review, cannot peer review another librarian’s work unless asked to do so, and must receive buy-in from the review team to include grey literature searches.

While nearly every survey respondent indicated that they intended to ask for authorship in the future, less than 60% of librarians involved in a systematic review after the class reported that they had asked for authorship, which was dismaying, especially given the 70% success rate of those who did ask. Attendees with no history of working on systematic reviews prior to the class were more likely than not to ask for authorship after class. Librarians who had worked on a systematic review prior to attending the class showed consistency in authorship behavior over time: those who had asked for authorship in the past were likely to ask for authorship after our class, while those who had not previously asked were no more likely to ask after attending the class.

This survey did not measure all individual and institutional factors that are associated with the likelihood of asking for authorship. Additional research is needed to identify and address these factors, but some information on possible contributing factors exists [15]. A survey of health sciences library administrators found high support for librarians’ involvement in systematic reviews but a lack of consensus about how much involvement would be required to merit coauthorship. It is possible that this uncertainty about librarian coauthorship is communicated to frontline librarians, leaving them unsure or hesitant about asking for authorship.

Creation of a formal systematic review service in a library may be one method for addressing this confusion. Such services can provide guidance or education on the intellectual contributions of librarians to reviews through establishing a formal policy [16, 17] or by requiring a memorandum of understanding [7]. It may also be helpful to provide librarians themselves with detailed training on standards for authorship, training on how their work on a review fits within those standards, and practical training on how to negotiate for authorship. Finally, Ross-White found that librarians were more likely to be coauthors on systematic reviews originating from departments or schools where librarians had a high level of previous outreach, which suggests that outreach to patrons, through either liaison librarians or classes about systematic reviews, can increase the number of librarians serving as coauthors [18].

In addition to observing changes in individual librarian practices, there appeared to be institutional changes occurring over the period of time covered by our study. At pre-class, less than one-third of librarians reported that their libraries had a systematic review service in place, and less than half were actively promoting the service. Six months after attending the class, there appeared to be an increase in the number of libraries offering such a service, while those with existing programs were changing or improving them. Among librarians reporting change in an institution’s systematic review service, many attributed the changes to the individual’s attendance at the class.

Finally, we explored librarians’ perceived barriers to participation in a systematic review project. Prior to attending the class, the most frequently endorsed barrier was their own lack of knowledge. At follow-up, this was the barrier least likely to be reported. The reduction of librarian knowledge as a barrier is further supported by the rise in knowledge test scores at post-test and maintenance of it at follow-up. The barriers that were most frequently reported at follow-up (researchers’ lack of knowledge or researchers’ failure to ask for librarian assistance) could be addressed by knowledgeable librarians offering classes to patrons on the basics of systematic review methodology or the role of comprehensive literature searching in reviews or through promoting librarian involvement in systematic reviews with well-publicized library systematic review services. Inexperienced researchers who request systematic review searches could be directed to sound methodology sources and be required to educate themselves before the librarian invests time in their projects. Library administrators may also want to discuss with other academic departments the possibility of developing systematic review methodology courses that could be integrated into the standard curriculum and offered to students on a for-credit basis.

Over time, changes in librarian behaviors and attitudes such as those reported in this study could contribute to a cultural shift in academic librarianship, with librarians moving from their traditional supporting role to a role better described as collegial or collaborative. Librarians who are comfortable with and capable in their role as coinvestigators on a systematic review may be well positioned to expand their participation to other types of research projects that utilize evidence-based approaches to address health topics or problems. This cultural shift may impact the entire practice of librarianship, changing training expectations for those who participate in research and possibly resulting in a new specialty. Library administrators may want to consider the implications of such a shift, such as how it might affect decisions about developing and maintaining the traditional core skills of librarians.

### Limitations and future evaluation considerations

This study has several limitations. Participants in the class were primarily medical librarians who had the time, resources, and financial support to travel to a two-and-a-half-day class and who arrived at the class already relatively knowledgeable about systematic reviews. Thus, our findings might not be generalizable to librarians who are from other specialty areas, who are newer to systematic review methodology, or who work in environments that provide less support or opportunity for CE training. Lack of a comparison or control group limits the causal conclusions that can be drawn. As noted earlier, “systematic review librarian” is an emerging role for health sciences librarians. Thus, the profession as a whole could be becoming more generally knowledgeable about and involved in systematic reviews over time. Finally, the observed retention of knowledge well after the end of the class could be due to additional training or CE activities engaged in by some of our respondents.

Kirkpatrick and Kirkpatrick noted that all training programs share the objective of increasing the knowledge of participants and that knowledge change is most valuable when it leads to positive change in participants’ behavior and organizational results [5]. Thus, the optimal program evaluation prospectively assesses the knowledge, skills, or behaviors that are expected to change as a result of participation in the program and the changes that occur. This type of evaluation can produce valuable information and feedback but is more complex than a one-time assessment of participant satisfaction and learning, requiring creation of questionnaires that assess knowledge application and professional behaviors at several different points in time.

Statistical analysis and interpretation are also more complex than for one-time surveys. Because of the time and effort required, prospective evaluation of training impact should be reserved for trainings of high importance, and outcomes that are measured should be carefully chosen with input from experienced methodologists and a statistician. Our experience suggests that for highly important training initiatives, this can be worth the effort. Trainers can use the information to improve the training and, through disseminating the results, provide others with valuable information on the design and implementation of professional workshops.

This longitudinal, prospective survey adds to knowledge about librarian instruction by demonstrating that librarians who are motivated by a desire to increase their knowledge about systematic reviews and to improve their systematic review skills can enhance their knowledge of systematic reviews, confidence in collaborating on systematic reviews, and engagement in systematic-review related behaviors by attending a rigorous, face-to-face CE course. We view our study as a first step in the process of understanding the impact CE classes can have on the knowledge and skills of librarians. Additional studies are needed to explore topics that were not addressed by our findings, such as the impact of class format (face-to-face, online, flipped) on librarian outcomes, optimal approaches for teaching core skills such as advanced literature searching, and best methods for measuring changes in skills and knowledge over time.

## SUPPLEMENTAL FILES

Appendix A Pre-class survey

Appendix B Post-class survey

Appendix C Follow-up survey

Appendix D Tables 1–8

## 

**Barbara L. Folb**, folb@pitt.edu, https://orcid.org/0000-0001-5531-980X, Librarian Emeritus, Health Sciences Library System, University of Pittsburgh, Pittsburgh, PA

**Mary L. Klem**, klem@pitt.edu, https://orcid.org/0000-0003-3481-5737, Research and Instruction Librarian and Liaison to the School of Nursing, Health Sciences Library System, University of Pittsburgh, Pittsburgh, PA

**Ada O. Youk**, ayouk@pitt.edu, https://orcid.org/0000-0001-6912-9759, Associate Professor, Graduate School of Public Health, Department of Biostatistics, University of Pittsburgh, Pittsburgh, PA

**Julia J. Dahm**, jdahm@pitt.edu, https://orcid.org/0000-0001-5907-8759, Coordinator of Technology Integration Services, Health Sciences Library System, University of Pittsburgh, Pittsburgh, PA

**Meiqi He**, meh206@pitt.edu, Data Analyst, Department of Pharmacy and Therapeutics, University of Pittsburgh, Pittsburgh, PA

**Andrea M. Ketchum**, ketchum@pitt.edu, https://orcid.org/0000-0002-4384-1294, Research and Instruction Librarian and Scholarly Communication Liaison, Health Sciences Library System, University of Pittsburgh, Pittsburgh, PA

**Charles B. Wessel**, cbw@pitt.edu, https://orcid.org/0000-0002-5018-0156, Head of Research Initiative, Health Sciences Library System, University of Pittsburgh, Pittsburgh, PA

**Linda M. Hartman, AHIP**, linda@hartman-team.com, https://orcid.org/0000-0003-0309-4463, Glenshaw, PA
