# Supplementary material for: Continuing education for systematic reviews: a prospective longitudinal assessment of a workshop for librarians
Source: J Med Libr Assoc. 2020 Jan 1;108(1):36–46. doi: 10.5195/jmla.2020.492 (PMC6919982; doi:10.5195/jmla.2020.492)
Supplement: Appendix A [file jmla-108-36-s001.pdf]

Journal of the Medical Library Association

Supplemental content to
J Med Libr Assoc. Jan;108(1):dx.doi.org/10.5195/jmla.2020.492
www.jmla.mlanet.org
© Folb, Klem, Youk, Dahm, He, Ketchum, Wessel, Hartman 2020

Continuing education for systematic reviews: a prospective longitudinal assessment of a workshop for librarians

Barbara L. Folb; Mary L. Klem; Ada O. Youk; Julia J. Dahm; Meiqi He; Andrea M. Ketchum; Charles B. Wessel; Linda M. Hartman, AHIP

APPENDIX A

Pre-class survey

University of Pittsburgh
Health Sciences Library System

Pre class Survey

The purpose of this research study is to examine the impact of attendance at a continuing education workshop on librarians. This is the first survey in a series of three: pre class, post class, and six month follow up. You are under no obligation to participate, and your decision about participation in the study will have no impact on your involvement in the workshop. There is no benefit or anticipated harm to you associated with taking this survey, and you have a right to withdraw from the study at any point.

This survey asks questions on characteristics of your workplace and the nature of your work, and professional practice and knowledge related to systematic reviews. The data we receive will be used for research purposes. Your responses remain confidential as the results will sit on a password protected server.

NOTE ON TERMINOLOGY: We use the 'library user' in this survey to refer to any library user who is participating in a systematic review. This can include clinicians, students, staff or researchers who use your library.
It will take about 10- 20 minutes to complete this survey.

Institutional Characteristics Questions

This section asks about the place where you currently work

***1. Where do you work? Please choose the most accurate description.**
Choose one of the following answers

- ○ Academic health sciences library
- ○ Academic library, not health sciences
- ○ Hospital library
- ○ Government library
- ○ Corporate library
- ○ Not currently working
- ○ Other: ____________

? If other, please describe.

*2. **How many staff members at your library or library system provide health sciences literature searching services? If you do not know the exact number, make your best estimate.**
Choose one of the following answers

Please choose...

Question 2 answer choices: 1; 2; 3; 4; 5; 6; 7; 8; 9; 10; 11; 12; 13; 14; 15

*3. **Does your library have a formal systematic review consultation service?**
Choose one of the following answers

- ○ Yes
- ○ No
- ○ Don't know

Participants only see Question 4 if the following conditions are met: Answer was "Yes" at Question 3: Does your library have a formal systematic review consultation service?

*4. **How many years has your systematic review service existed? If you do not know the exact number, make your best estimate.**
Choose one of the following answers

Please choose...

Question 4 answer choices: 1; 2; 3; 4; 5; 6; 7; 8; 9; 10; 11; 12; 13; 14; 15

Participants only see Question 5 if the following conditions are met: Answer was "No" or "Don't Know" at Question 3: Does your library have a formal systematic review consultation service?

*5. **Does your library have plans to implement one in the near future?**

○ Yes ○ No

*6. **Does your library promote systematic review searching services to your library users?**

○ Yes ○ No

Participants only see Question 7 if the following conditions are met: Answer was "Yes" at Question 6: Does your library promote systematic review searching services to your library users?

7. What methods does your library use to promote systematic review searching services at your institution? Please check all that apply.

☐ Website

☐ Newsletter

☐ Other, Please describe: ________

*

For questions 8 and 10, please indicate your degree of agreement with the following statements, where 1 is strongly agree and 5 is strongly disagree

8. Administrators at my library enthusiastically support librarian involvement in systematic review searching.

1 - Strongly Agree	2 - Agree	3 - Neutral	4 - Disagree	5 - Strongly Disagree	0 - Don't Know
○	○	○	○	○	○

9. Comments on question 8

*10. Researchers, clinicians, and staff (exclude students from this question) in my workplace who do systematic reviews typically have librarians do the searching for systematic reviews.**

	1 - Strongly Agree	2 - Agree	3 - Neutral	4 - Disagree	5 - Strongly Disagree	6 - Not Applicable	0 - Don't Know
	○	○	○	○	○	○	○

11. Comments on question 10

*

12. Do students at your workplace do systematic reviews as part of class assignments or thesis or dissertation work?

Choose one of the following answers

- ○ Yes
- ○ No
- ○ Don't Know

Participants only see Question 13 if the following conditions are met: Answer was "Yes" at Question 12: Do students at your workplace do systematic review as part of class assignment or thesis or dissertation work?

***13. Do students engaged in systematic review searching at your institution consult with librarians or receive training from librarians?**

Choose one of the following answers

- ○ All of the time
- ○ Most of the time
- ○ Some of the time
- ○ Seldom
- ○ Never
- ○ Don't know

*14. What barriers, if any, have you encountered to being involved in systematic review searching? Check all that apply.

- ☐ Not enough time
- ☐ Not enough librarians on staff
- ☐ Lack of library administrative support
- ☐ Need to know more about systematic reviews
- ☐ Researchers don't ask
- ☐ Researchers don't understand the systematic review process
- ☐ There are more requests for systematic reviews than we can accommodate
- ☐ Other duties more pressing
- ☐ No barriers noted
- ☐ Other, fill in the blank: ________

Practice Characteristics Questions, Pretest

This section asks about your experiences and practices with working on systematic reviews.

*

15. Have you ever worked on a systematic review search? (Note: does not need to be complete to say yes).

○ Yes ○ No

Participants only see Question 16 if the following conditions are met: Answer was "Yes" at Question 15: Have you ever worked on a systematic review search?

*

16. How many systematic review projects have you provided the searches for? If you do not recall the exact number make your best estimate.

Choose one of the following answers

Please choose...

Question 16 answer choices: 0; 1; 2; 3; 4; 5; 6; 7; 8; 9; 10; 11; 12; 13; 14; 15; 16; 17; 18; 19; 20; 21; 22; 23; 24; 25; 26; 27; 28; 29; 30; 31; 32; 33; 34; 35; 36; 37; 38; 39; 40; 41; 42; 43; 44; 45; 46; 47; 48; 49; 50; More than 50

Participants only see Question 17 if the following conditions are met: Answer was "Yes" at Question 15: Have you ever worked on a systematic review search?

***17. In the past year have you sought peer review for a systematic review search that you completed?**

○ Yes ○ No

Participants only see Question 18 if the following conditions are met: Answer was "Yes" at Question 15: Have you ever worked on a systematic review search?

*

18. Have you ever searched for grey literature as part of a systematic review search?

Choose one of the following answers

○ Yes
○ No
○ Don't know

Participants only see Question 19 if the following conditions are met: Answer was "Yes" at Question 15: Have you ever worked on a systematic review search?

*

19. Have you ever asked for authorship on a systematic review?

○ Yes ○ No

Participants only see Question 20 if the following conditions are met: Answer was "Yes" at Question 15: Have you ever worked on a systematic review search? *and* Answer was "Yes" at Question 19: Have you ever asked for authorship on a systematic review?

*

20. Was your request for authorship successful?

Choose one of the following answers

○ Yes
○ No
○ Don't know yet

*

21. In the past year how many consultations have you provided to library users who wanted to do the systematic review search on their own? If you do not recall exactly, make your best estimate. Consultations could be on any aspects of systematic reviews (searching, file management, etc.) Multiple consults supporting a single systematic review count as more than one search.

Choose one of the following answers

Please choose...

Question 21 answer choices: 0; 1; 2; 3; 4; 5; 6; 7; 8; 9; 10; 11; 12; 13; 14; 15; 16; 17; 18; 19; 20; 21; 22; 23; 24; 25; 26; 27; 28; 29; 30; 31; 32; 33; 34; 35; 36; 37; 38; 39; 40; 41; 42; 43; 44; 45; 46; 47; 48; 49; 50; More than 50

22. **Indicate how much you agree or disagree with the following statement:**

As a consultant I can communicate to library users the nuances and subtleties of systematic review searching.

	1 - Strongly Agree	2 - Agree	3 - Neutral	4 - Disagree	5 - Strongly Disagree	0 - Don't Know	No answer
	○	○	○	○	○	○	◉

*

23. In the past year have you peer reviewed another librarian's systematic review search?

Choose one of the following answers

○ Yes
○ No
○ Don't know

*

24. Have you taken any other professional development workshops for the purpose of increasing your systematic review related skills or knowledge?

Choose one of the following answers

○ Yes
○ No
○ Don't know

*25. **Since becoming a librarian have you ever enrolled in a for-credit university class in any of the following subject areas relevant to systematic review skills? Please check all that apply**

- ☐ Statistics
- ☐ Epidemiology
- ☐ Research methods
- ☐ Evidence-based health (any discipline- medicine, nursing, public health etc)
- ☐ Health literature appraisal
- ☐ None noted
- ☐ Other relevant class: ____________

*

26. Have librarians at your home institution worked together in any of the following ways to increase their knowledge or skills about systematic reviews? Please check all that apply:

- ☐ Journal club
- ☐ Invited speakers
- ☐ Attended local workshop
- ☐ Other group study activity
- ☐ One on one mentoring
- ☐ None noted
- ☐ Other: ____________

*

27. I have read all or part of the Institute of Medicine (IOM) report *Finding What Works in Health Care: Standards for Systematic Reviews.*

Choose one of the following answers

- ○ Yes
- ○ No
- ○ Don't know

*

28. I use published guidelines (Cochrane, PRISMA, etc) when documenting systematic review search strategies.

	1 - Strongly Agree	2 - Agree	3 - Neutral	4 - Disagree	5 - Strongly Disagree	0 - Don't Know
	○	○	○	○	○	○

***29. I can complete a high quality systematic review search.**

	1 - Strongly Agree	2 - Agree	3 - Neutral	4 - Disagree	5 - Strongly Disagree
	○	○	○	○	○

Knowledge Questions

The following section asks seven questions about systematic reviews.

***30. You are beginning a systematic review search for a review is on the therapeutic management of carotid stenosis. Of the following databases, which three would you consider most important to search? Please check the boxes next to them.**

- ☐ CINAHL
- ☐ Embase
- ☐ PsycINFO
- ☐ MEDLINE
- ☐ Cochrane Central Register of Controlled Trials

For Question 30, please choose at most 3 answers.

*

31. Rank the following studies from highest to lowest on the strength of evidence they would provide on a therapeutic question, with 1 being the highest and 4 being the lowest.

Click on an item in the list on the left, starting with your highest ranking item, moving through to your lowest ranking item.

Your choices:

- Cohort study
- Narrative review
- Systematic review
- Randomized controlled trial

Your ranking:

1: ________

2: ________

3: ________

4: ________

Click on the scissors next to each item on the right to remove the last entry in your ranked list

*

32. You are conducting a prognosis systematic review on the question: "Does smoking status (smoker vs. non-smoker) influence mortality risk in patients who have experienced an acute MI?" If the researcher requests the search be limited by study type, which of the following is the researcher most likely to request be included?

Choose one of the following answers

○ An experimental study design such as: Single blinded randomized controlled trial

○ An observational study design such as: cohort study

*

For the next 3 questions, indicate whether each statement about publication bias is true or false.

33. Publication bias is what occurs when only English language publications are used

Choose one of the following answers

○ True

○ False

*

34. Publication bias is what occurs whenever the research that appears in the published literature is systematically unrepresentative of the population of complete studies.

Choose one of the following answers

- ◯ True
- ◯ False

*

35. Publication bias is what occurs when only studies done in the United States are used.

Choose one of the following answers

- ◯ True
- ◯ False

*

For the next 3 questions indicate whether each statement about search filters is true or false:

36. Search filters are pre-determined sets of search terms.

Choose one of the following answers

- ◯ True
- ◯ False

*

37. All search filters have been formally evaluated and validated.

Choose one of the following answers

- ○ True
- ○ False

*

38. A validated filter that has been edited or changed in some way is still considered a validated filter.

Choose one of the following answers

- ○ True
- ○ False

***39. Which of the following is true about grey literature?**

Choose one of the following answers

- ○ A. Grey literature is unpublished literature only
- ○ B. Inclusion of grey literature in a systematic review does not reduce publication bias
- ○ C. Both A and B
- ○ D. Neither A or B

*

40. According to PRISMA standards, which of the following must be included in the Methods section of a systematic review manuscript? Please check all that apply.

- ☐ Database and platform
- ☐ The complete search strategy used in every database
- ☐ Start and end date of each database search
- ☐ The individual responsible for conducting the literature searches
- ☐ I am not familiar with PRISMA

Motivation Question

41. What is the most important reason you are here today?

Work Questions

The last section asks for employment information how much time you spend on specific types of library work.

***42. Choose the category that most accurately represents your current job.**

- ○ User services (for example: education, reference, liaison, informationist, researcher etc)
- ○ Management (for example: administrator, director, assistant director)
- ○ Outreach (services to populations outside of your home institution)
- ○ Technical services (for example, cataloging, systems, digital librarian)
- ○ Other:

*

43. What percent of your work time is devoted to reference work such as answering questions, teaching, and working on searches?

Choose one of the following answers

Please choose...

Question 43 answer choices: 0%; 1-20%; 21-40%; 41-60%; 61-80%; 81-100%

*

44. What percent of your work time is devoted to administrative work such as supervising others, budget management, and planning?

Choose one of the following answers

Please choose...

Question 44 answer choices: 0%; 1-20%; 21-40%; 41-60%; 61-80%; 81-100%

***45. How many years have you worked as a librarian?**

Choose one of the following answers

Please choose...

Question 45 answer choices: 0; 1; 2; 3; 4; 5; 6; 7; 8; 9; 10; 11; 12; 13; 14; 15; 16; 17; 18; 19; 20; 21; 22; 23; 24; 25; 26; 27; 28; 29; 30; 31; 32; 33; 34; 35; 36; 37; 38; 39; 40

***46. Which of the following degrees have you earned? Please check all that apply.**

- ☐ Bachelor's degree or equivalent. List subject area(s):
- ☐ MLS, MLIS or equivalent
- ☐ Masters degree or equivalent, in a subject other than library science. List subject area(s):
- ☐ Doctorate degree (for example PhD, EdD, etc). List subject area(s):
- ☐ Professional degree beyond a bachelor's degree (for example MD, DDS, LLB, JD or equivalent). List subject area(s):
