# Supplementary material for: Continuing education for systematic reviews: a prospective longitudinal assessment of a workshop for librarians
Source: J Med Libr Assoc. 2020 Jan 1;108(1):36–46. doi: 10.5195/jmla.2020.492 (PMC6919982; doi:10.5195/jmla.2020.492)
Supplement: Appendix B [file jmla-108-36-s002.pdf]

JMLA Journal of the Medical Library Association

Supplemental content to
J Med Libr Assoc. Jan;108(1):dx.doi.org/10.5195/jmla.2020.492
www.jmla.mlanet.org
© Folb, Klem, Youk, Dahm, He, Ketchum, Wessel, Hartman 2020

Continuing education for systematic reviews: a prospective longitudinal assessment of a workshop for librarians

Barbara L. Folb; Mary L. Klem; Ada O. Youk; Julia J. Dahm; Meiqi He; Andrea M. Ketchum; Charles B. Wessel; Linda M. Hartman, AHIP

APPENDIX B

Post-class survey

University of Pittsburgh
Health Sciences Library System

Post Class Survey

The purpose of this research study is to examine the impact of attendance at a continuing education workshop on librarians. This is the second survey in a series of three: pre class, post class, and six month follow up. You are under no obligation to participate, and your decision about participation in the study will have no impact on your involvement in the workshop. There is no benefit or anticipated harm to you associated with taking this survey, and you have a right to withdraw from the study at any point.

This survey asks questions on characteristics of your workplace and the nature of your work, and professional practice and knowledge related to systematic reviews. The data we receive will be used for research purposes. Your responses remain confidential as the results will sit on a password protected server.

NOTE ON TERMINOLOGY: We use the library user in this survey to refer to any library user who is participating in a systematic review. This can include clinicians, students, staff or researchers who use your library.

It will take about 5-10 minutes to complete this survey.

There are 37 questions in this survey

Practice Characteristics Questions, Posttest

Please indicate your degree of agreement with the following statements, where 1 is strongly agree and 5 is strongly disagree.

*1. Having taken the systematic review class I am more likely to work on systematic review searches in the future.

1 - Strongly Agree	2 - Agree	3 - Neutral	4 - Disagree	5 - Strongly Disagree	0 - Don't Know
○	○	○	○	○	○

2. Comments on question 1

*3. Having taken the class, I am <u>more</u> likely to provide systematic review consultations to library users at my institution.

1 - Strongly Agree	2 - Agree	3 - Neutral	4 - Disagree	5 - Strongly Disagree	0 - Don't Know
○	○	○	○	○	○

4. Comment on question 3

*5. As a consultant I can communicate to library users the nuances and subtleties of systematic review searching.

1 - Strongly Agree	2 - Agree	3 - Neutral	4 - Disagree	5 - Strongly Disagree	0 - Don't Know
○	○	○	○	○	○

6. Comment on question 5

*7. Having taken the class, I am more likely to promote peer review for systematic review searches at my library.

1 - Strongly Agree	2 - Agree	3 - Neutral	4 - Disagree	5 - Strongly Disagree	0 - Don't Know
○	○	○	○	○	○

8. Comment on question 7

*9. Having taken the class, I am <u>less</u> likely to seek peer review for my own systematic review searches.

	1 - Strongly Agree	2 - Agree	3 - Neutral	4 - Disagree	5 - Strongly Disagree	0 - Don't Know
	○	○	○	○	○	○

10. Comment on question 9

*11. Having taken the class, I intend to read all or part of the IOM standards for systematic reviews.

	1 - Strongly Agree	2 - Agree	3 - Neutral	4 - Disagree	5 - Strongly Disagree	0 - Don't Know
	○	○	○	○	○	○

12. Comment on question 11

*13. Having taken the class, I intend to use published guidelines (Cochrane, PRISMA, etc) when documenting systematic review search strategies.

	1 - Strongly Agree	2 - Agree	3 - Neutral	4 - Disagree	5 - Strongly Disagree	0 - Don't Know
	○	○	○	○	○	○

14. Comment on question 13

*15. Having taken the class, I am <u>less</u> likely to suggest searching of grey literature resources to library users as part of a systematic review search.

	1 - Strongly Agree	2 - Agree	3 - Neutral	4 - Disagree	5 - Strongly Disagree	0 - Don't Know
	○	○	○	○	○	○

16. Comment on question 15

*17. Having taken the class, I intend to ask for authorship on systematic reviews.

	1 - Strongly Agree	2 - Agree	3 - Neutral	4 - Disagree	5 - Strongly Disagree	0 - Don't Know
	○	○	○	○	○	○

18. Comment on question 17

*19. I will use the materials on the USB drive after the workshop.

	1 - Strongly Agree	2 - Agree	3 - Neutral	4 - Disagree	5 - Strongly Disagree	0 - Don't Know
	○	○	○	○	○	○

20. Comment on question 19

*21. In the future, I do not plan to attend other professional development workshops on systematic review topics.

	1 - Strongly Agree	2 - Agree	3 - Neutral	4 - Disagree	5 - Strongly Disagree	0 - Don't Know
	○	○	○	○	○	○

22. Comment on question 21

*23. I intend to share the information from this class with my colleagues.

	1 - Strongly Agree	2 - Agree	3 - Neutral	4 - Disagree	5 - Strongly Disagree	0 - Don't Know
	◯	◯	◯	◯	◯	◯

24. Comment on question 23

*25. I can complete a high quality systematic review search.

	1 - Strongly Agree	2 - Agree	3 - Neutral	4 - Disagree	5 - Strongly Disagree
	◯	◯	◯	◯	◯

26. Comment on question 25

Knowledge Questions

The following section asks seven questions about systematic reviews.

*27. You are beginning a systematic review search for a review on the therapeutic management of carotid stenosis. Of the following databases, which three would you consider most important to search? Please check the boxes next to them.

- ☐ CINAHL
- ☐ Embase
- ☐ PsycINFO
- ☐ MEDLINE
- ☐ Cochrane Central Register of Controlled Trials

For Question 27, please choose at most 3 answers.

*28. Rank the following studies from highest to lowest on the strength of evidence they would provide on a therapeutic question, with 1 being the highest and 4 being the lowest.

Click on an item in the list on the left, starting with your highest ranking item, moving through to your lowest ranking item.

Your choices:

- Cohort study
- Narrative review
- Systematic review
- Randomized controlled trial

Your ranking:

1:

2:

3:

4:

Click on the scissors next to each item on the right to remove the last entry in your ranked list

*29. You are conducting a prognosis systematic review on the question: "Does smoking status (smoker vs. non-smoker) influence mortality risk in patients who have experienced an acute MI?" If the reviewer requests the search be limited by study type, which of the following is the researcher most likely to request be included?

Choose one of the following answers

- ○ An experimental study design such as: Single blinded randomized controlled trial
- ○ An observational study design such as: cohort study

*For the next 3 questions, indicate whether each statement about publication bias is true or false.

30. Publication bias occurs when only English language publications are used.
Choose one of the following answers

- True
- False

*31. Publication bias occurs when the research that appears in the published literature is systematically unrepresentative of the population of complete studies.
Choose one of the following answers

- True
- False

*32. Publication bias occurs when only studies done in the United States are used.
Choose one of the following answers

- True
- False

*For the next 3 questions indicate whether each statement about search filters is true or false:

33. Search filters are pre-determined sets of search terms.
Choose one of the following answers

- True
- False

*34. All search filters have been formally evaluated and validated.
Choose one of the following answers

- True
- False

*35. A validated filter that has been edited or changed in some way is still considered a validated filter.
Choose one of the following answers

- True
- False

*36. Which of the following is true about grey literature?
Choose one of the following answers

- A. Grey literature is unpublished literature only
- B. Inclusion of grey literature in a systematic review does not reduce publication bias
- C. Both A and B
- D. Neither A or B

*37. According to PRISMA standards, which of the following must be included in the Methods section of a systematic review manuscript? Please check all that apply.

- [ ] Database and platform
- [ ] The complete search strategy used in every database
- [ ] Start and end date of each database search
- [ ] The individual responsible for conducting the literature searches
- [ ] I am not familiar with PRISMA
