# Supplementary material for: Continuing education for systematic reviews: a prospective longitudinal assessment of a workshop for librarians
Source: J Med Libr Assoc. 2020 Jan 1;108(1):36–46. doi: 10.5195/jmla.2020.492 (PMC6919982; doi:10.5195/jmla.2020.492)
Supplement: Appendix C [file jmla-108-36-s003.pdf]

JMLA Journal of the Medical Library Association

Supplemental content to
J Med Libr Assoc. Jan;108(1):dx.doi.org/10.5195/jmla.2020.492
www.jmla.mlanet.org
© Folb, Klem, Youk, Dahm, He, Ketchum, Wessel, Hartman 2020

Continuing education for systematic reviews: a prospective longitudinal assessment of a workshop for librarians

Barbara L. Folb; Mary L. Klem; Ada O. Youk; Julia J. Dahm; Meiqi He; Andrea M. Ketchum; Charles B. Wessel; Linda M. Hartman, AHIP

APPENDIX C

Follow-up survey

University of Pittsburgh
HSLS Health Sciences Library System

Follow Up Survey Copy

The purpose of this research study is to examine the impact of attendance at a continuing education workshop on librarians. This is the final survey in a series of three: pre class, post class, and six month follow up. You are under no obligation to participate in this survey. There is no benefit or anticipated harm to you associated with taking this survey, and you have a right to withdraw from the study at any point.

This survey asks questions about characteristics of your workplace and the nature of your work, and professional practice and knowledge related to systematic reviews. The data we receive will be used for research purposes. Your responses remain confidential as the results will sit on a password protected server.

NOTE ON TERMINOLOGY. We use the word "library user" in this survey to refer to any library user who is participating in a systematic review. This can include clinicians, students, staff or researchers who use your library.

It will take about 10-15 minutes to complete the survey.

There are 48 questions in this survey.

Work Questions

***1. Has your job title changed since you took the class?**

○ Yes ○ No

Participants only see Question 2 if the following conditions are met: Answer was "Yes" at Question 1: Has your job title changed since you took the class?

***2. What is your current job title?**

***3. Has your place of employment changed since you took the systematic review workshop?**

○ Yes ○ No

Institutional Characteristics Questions

This section asks about the place where you currently work

Participants only see Question 4 if the following conditions are met: Answer was "Yes" at Question 3: Has your place of employment changed since you took the systematic review workshop?

***4. Where do you currently work?**

Please choose the most accurate description.

- Academic health sciences library
- Academic library, not health sciences
- Hospital library
- Government library
- Corporate library
- Not currently working
- Other: ____

? If other, please describe.

Participants only see Question 5 if the following conditions are met: Answer was "Yes" at Question 3: Has your place of employment changed since you took the systematic review workshop?

***5. How many staff members at your library or library system provide health sciences literature searching services?**

If you do not know the exact number, make your best estimate.

Choose one of the following answers

Please choose...

Question 5 answer choices: 1; 2; 3; 4; 5; 6; 7; 8; 9; 10; 11; 12; 13; 14; 15

***6. Since you attended the University of Pittsburgh systematic review course have there been any changes to the systematic review services offered at your institution?**

- Yes
- No
- Don't know

Participants only see Question 7 if the following conditions are met: Answer was "Yes" at Question 6: Since you attend the University of Pittsburgh systematic review course have there been any changes to the systematic review services offered at your institution?

***7. Did your library add a formal systematic review service?**

- Yes
- No
- Not applicable; already had one

Participants only see Question 8 if the following conditions are met: Answer was "Yes" at Question 6: Since you attend the University of Pittsburgh systematic review course have there been any changes to the systematic review services offered at your institution?

8. Please describe any other changes to your systematic review services.

Participants only see Question 9 if the following conditions are met: Answer was "Yes" at Question 6: Since you attend the University of Pittsburgh systematic review course have there been any changes to the systematic review services offered at your institution?

***9. In your opinion, to what degree did the University of Pittsburgh systematic review course contribute to the changes to your library's systematic review services?**

1 - To a great degree	2 - To some degree	3 - Neutral	4 - To a minimal degree	5 - Not at all	0 - Don't Know
○	○	○	○	○	○

***10. Does your library promote systematic review searching services to your library users?**

○ Yes ○ No

Participants only see Question 11 if the following conditions are met: Answer was "Yes" at Question 10: Does your library promote systematic review searching services to your library users?

11. What methods does your library use to promote systematic review searching services at your institution?

Please check all that apply.

- ☐ Website
- ☐ Newsletter
- ☐ Other, Please describe: ______

*Please indicate your degree of agreement with the following statements, where 1 is strongly agree and 5 is strongly disagree.

12. Administrators at my library enthusiastically support librarian involvement in systematic review searching.

1 - Strongly Agree	2 - Agree	3 - Neutral	4 - Disagree	5 - Strongly Disagree	0 - Don't Know
○	○	○	○	○	○

13. Comments on 12.

***14. Researchers, clinicians, and staff (exclude students from this question) in my workplace who do systematic reviews typically have librarians do the searching for systematic reviews.**

1 - Strongly Agree	2 - Agree	3 - Neutral	4 - Disagree	5 - Strongly Disagree	0 - Don't Know
○	○	○	○	○	○

15. Comments on 14.

***16. What barriers, if any, have you encountered to being involved in systematic review searching?**

Check all that apply.

- ☐ Not enough time
- ☐ Not enough librarians on staff
- ☐ Lack of library administrative support
- ☐ Need to know more about systematic reviews
- ☐ Library users don't ask
- ☐ Library users don't understand the systematic review process
- ☐ There are more requests for systematic reviews than we can accommodate
- ☐ Other duties more pressing
- ☐ No barriers noted
- ☐ Other, fill in the blank: ________

Practice Characteristics Questions, Pretest

This section asks about your experiences and practices with working on systematic reviews.

***17. Have you worked on any systematic review searches since completing the University of Pittsburgh Systematic Review class? (Note: does not need to be complete to select yes).**

○ Yes ○ No

Participants only see Question 18 if the following conditions are met: Answer was "Yes" at Question 17: Have you worked on any systematic review searches since completing the University of Pittsburgh Systematic Review class?

***18. How many systematic review searches have you worked on since completing the class?**

If you do not recall the exact number make your best estimate.

Please choose... ▾

Question 18 answer choices: 0; 1; 2; 3; 4; 5; 6; 7; 8; 9; 10; 11; 12; 13; 14; 15; 16; 17; 18; 19; 20; 21; 22; 23; 24; 25; 26; 27; 28; 29; 30; 31; 32; 33; 34; 35; 36; 37; 38; 39; 40; 41; 42; 43; 44; 45; 46; 47; 48; 49; 50

Participants only see Question 19 if the following conditions are met: Answer was "Yes" at Question 17: Have you worked on any systematic review searches since completing the University of Pittsburgh Systematic Review class?

***19. Since taking the class have you sought peer review for a systematic review search that you completed?**

○ Yes ○ No

Participants only see Question 20 if the following conditions are met: Answer was "Yes" at Question 17: Have you worked on any systematic review searches since completing the University of Pittsburgh Systematic Review class?

***20. Since taking the class have you searched for grey literature as part of a systematic review search?**

- Yes
- No
- Don't know

Participants only see Question 21 if the following conditions are met: Answer was "Yes" at Question 17: Have you worked on any systematic review searches since completing the University of Pittsburgh Systematic Review class?

***21. Since taking the class have you asked for authorship on a systematic review?**

- Yes
- No

Participants only see Question 22 if the following conditions are met: Answer was "Yes" at Question 17: Have you worked on any systematic review searches since completing the University of Pittsburgh Systematic Review class? *and* Answer was "Yes" at Question 21: Since taking the class have you asked for authorship on a systematic review?

***22. Was your request for authorship successful?**

- Yes
- No
- Don't know yet

***23. Since completing the class, how many consultations on systematic review search methods have you provided to researchers who wanted to do the systematic review search on their own? If you do not recall exactly, make your best estimate. Consultations could be on any aspects of systematic reviews (searching, file management, etc.) Multiple consults supporting a single systematic review count as more than one consultation.**

Please choose...

Question 23 answer choices: 0; 1; 2; 3; 4; 5; 6; 7; 8; 9; 10; 11; 12; 13; 14; 15; 16; 17; 18; 19; 20; 21; 22; 23; 24; 25; 26; 27; 28; 29; 30; 31; 32; 33; 34; 35; 36; 37; 38; 39; 40; 41; 42; 43; 44; 45; 46; 47; 48; 49; 50

***24. Indicate how much you agree or disagree with the following statement:**

As a consultant I can communicate to library users the nuances and subtleties of systematic review searching.

	1 - Strongly Agree	2 - Agree	3 - Neutral	4 - Disagree	5 - Strongly Disagree	0 - Don't Know
	○	○	○	○	○	○

***25. Since completing the class, have you peer reviewed another librarian's systematic review search?**

- ○ Yes
- ○ No
- ○ Don't know

***26. Have you used the course materials that you received on the USB drive?**

- ○ Yes
- ○ No
- ○ Don't recall

27. Do you have any feedback about the materials provided?

*28. **Since completing the class, have you taken any other professional development workshops for the purpose of increasing your systematic review related skills or knowledge?**

- Yes
- No
- Don't know

*29 **Since completing the class, have you enrolled in a for-credit university class in any of the following subject areas relevant to systematic review skills?**

Please check all that apply.

- Statistics
- Epidemiology
- Research methods
- Evidence-based health (any discipline- medicine, nursing, public health etc)
- Health literature appraisal
- None noted
- Other relevant class:

***30. Since completing the class, have librarians at your home institution worked together in any of the following ways to increase their knowledge or skills about systematic reviews? Please check all that apply:**

- ☐ Journal club
- ☐ Invited speakers
- ☐ Attended local workshop
- ☐ Other group study activity
- ☐ One on one mentoring
- ☐ None noted
- ☐ Other: ____________

***31. Since completing the class, have you shared the information from the systematic review class with your colleagues?**

○ Yes ○ No

Participants only see Question 32 if the following conditions are met: Answer was "Yes" at Question 31. Since completing the class, have you shared the information from the systematic review class with your colleagues?

***32. Please describe how you have shared the materials.**

____

33. I have read all or part of the Institute of Medicine (IOM) report *Finding What Works in Health Care: Standards for Systematic Reviews.

- ○ Yes
- ○ No
- ○ Don't know

*34. I use published guidelines (Cochrane, PRISMA, etc) when documenting systematic review search strategies.

1 - Strongly Agree	2 - Agree	3 - Neutral	4 - Disagree	5 - Strongly Disagree	0 - Not Applicable
○	○	○	○	○	○

*35. I can complete a high quality systematic review search.

1 - Strongly Agree	2 - Agree	3 - Neutral	4 - Disagree	5 - Strongly Disagree
○	○	○	○	○

Knowledge Questions

The following section asks seven questions about systematic reviews.

*36. You are beginning a systematic review search for a review on the therapeutic management of carotid stenosis.

Of the following databases, which three would you consider most important to search?

Please check the boxes next to them.

- ☐ CINAHL
- ☐ Embase
- ☐ PsycINFO
- ☐ MEDLINE
- ☐ Cochrane Central Register of Controlled Trials

For Question 36, please choose at most 3 answers.

*37. Rank the following studies from highest to lowest on the strength of evidence they would provide on a therapeutic question, with 1 being the highest and 4 being the lowest.**

Click on an item in the list on the left, starting with your highest ranking item, moving through to your lowest ranking item.

Your choices:

Cohort study
Narrative review
Randomized controlled trial
Systematic review

Your ranking:

1:
2:
3:
4:

Click on the scissors next to each item on the right to remove the last entry in your ranked list

*38. You are conducting a prognosis systematic review on the question: "Does smoking status (smoker vs. non-smoker) influence mortality risk in patients who have experienced an acute MI?"**

If the researcher requests the search be limited by study type, which of the following is the researcher most likely to request be included?

Please choose **only one** of the following.

- An experimental study design such as: Single blinded randomized controlled trial
- An observational study design such as: cohort study

*For the next 3 questions, indicate whether each statement about publication bias is true or false.

39. Publication bias occurs when only English language publications are used.

Please choose **only one** of the following:

- True
- False

*40. **Publication bias occurs when the research that appears in the published literature is systematically unrepresentative of the population of complete studies.**

Please choose **only one** of the following:

- True
- False

*41. **Publication bias occurs when only studies done in the United States are used.**

Please choose **only one** of the following:

- True
- False

*For the next 3 questions indicate whether each statement about search filters is true or false:

42. Search filters are pre-determined sets of search terms.

- True
- False

*43. **All search filters have been formally evaluated and validated.**

- True
- False

***44. A validated filter that has been edited or changed in some way is still considered a validated filter.**

- True
- False

***45. Which of the following is true about grey literature?**

Choose one of the following answers

- A. Grey literature is unpublished literature only
- B. Inclusion of grey literature in a systematic review does not reduce publication bias
- C. Both A or B
- D. Neither A or B

***46. According to PRISMA standards, which of the following must be included in the Methods section of a systematic review manuscript?**

Please check all that apply.

- [ ] Database and platform
- [ ] The complete search strategy used in every database
- [ ] Start and end date of each database search
- [ ] The individual responsible for conducting the literature searches
- [ ] I am not familiar with PRISMA

Work Questions

The last section asks for employment information concerning how much time you spend on specific types of library work.

***47. What percent of your work time is devoted to reference work such as answering questions, teaching, and working on searches?**

Please choose...

Question 47 answer choices: 0%; 1-5%; 6-10%; 11-15%; 16-20%; 21-25%; 26-30%; 31-35%; 36-40%; 41-45%; 46-50%; 51-55%; 56-60%; 61-65%; 66-70%; 71-75%; 76-80%; 81-85%; 86-90%; 91-95%; 96-100%

***48. What percent of your work time is devoted to administrative work such as supervising others, budget management, and planning?**

Please choose...

Question 48 answer choices: 0%; 1-5%; 6-10%; 11-15%; 16-20%; 21-25%; 26-30%; 31-35%; 36-40%; 41-45%; 46-50%; 51-55%; 56-60%; 61-65%; 66-70%; 71-75%; 76-80%; 81-85%; 86-90%; 91-95%; 96-100%
