# Supplementary material for: Continuing education for systematic reviews: a prospective longitudinal assessment of a workshop for librarians
Source: J Med Libr Assoc. 2020 Jan 1;108(1):36–46. doi: 10.5195/jmla.2020.492 (PMC6919982; doi:10.5195/jmla.2020.492)
Supplement: Appendix D [file jmla-108-36-s0004.pdf]

JMLA Journal of the Medical Library Association

Supplemental content to
J Med Libr Assoc. Jan;108(1):dx.doi.org/10.5195/jmla.2020.492
www.jmla.mlanet.org
© Folb, Klem, Youk, Dahm, He, Ketchum, Wessel, Hartman 2020

Continuing education for systematic reviews: a prospective longitudinal assessment of a workshop for librarians

Barbara L. Folb; Mary L. Klem; Ada O. Youk; Julia J. Dahm; Meiqi He; Andrea M. Ketchum; Charles B. Wessel; Linda M. Hartman, AHIP

APPENDIX D

Tables

Table of contents

Table 1. Relationship between survey questions and concepts measured at pre-class, post-class, and follow-up 2
Table 2. Overall and individual survey response rates 4
Table 3. Librarian characteristics, pre-class survey 5
Table 4. Workplace characteristics, pre-class survey 5
Table 5. Behavioral changes from pre-class to follow-up 6
Table 6. Reported and intended systematic review educational activities, pre-class, post-class, and follow-up 6
Table 7. Staff systematic review education activities in the workplace, pre-class and follow-up 7
Table 8. Pre-class existence of formal systematic review service, plans for future service, and changes at follow-up 8

JMLA Journal of the Medical Library Association

Supplemental content to
J Med Libr Assoc. Jan;108(1):dx.doi.org/10.5195/jmla.2020.492
www.jmla.mlanet.org
© Folb, Klem, Youk, Dahm, He, Ketchum, Wessel, Hartman 2020

Table 1 Relationship between survey questions and concepts measured at pre-class, post-class, and follow-up

Survey section	Pre-class (Appendix A) 46 questions total	Post-class (Appendix B) 37 questions total	Follow-up (Appendix C) 48 questions total
**Only measured at pre-class**			
Work questions	Librarian characteristics (45–46)*		
Motivation question	Motivation for attending workshop (41)		
**Measured at pre-class and follow-up**			
Work questions	Job characteristics (42–44)		Job characteristics (2, 47, 48)
Institutional characteristics	Workplace type (1, 2)		Workplace type (4, 5)
Institutional characteristics	Workplace systematic review services (3–5)		Workplace systematic review service changes (6, 7, 8)
Institutional characteristics	Promotion of systematic review services (6, 7)		Promotion of systematic review services (10, 11)
Institutional characteristics	Administrator support for systematic review services (8, 9)		Administrator support for systematic review services (12, 13)
Institutional characteristics	Patron use of systematic review services (10–13)		Patron use of systematic review services (14, 15)
Institutional characteristics	Barriers to systematic review involvement (14)	`	Barriers to systematic review involvement (16)
**Only measured at follow-up**			
Work questions			Job title, place changes (1, 3)
Institutional characteristics			Contribution of workshop to workplace systematic review service changes (9)

JMLA Journal of the Medical Library Association

Supplemental content to
J Med Libr Assoc. Jan;108(1):dx.doi.org/10.5195/jmla.2020.492
www.jmla.mlanet.org
© Folb, Klem, Youk, Dahm, He, Ketchum, Wessel, Hartman 2020

Table 1 Relationship between survey questions and concepts measured at pre-class, post-class, and follow-up (continued)

Survey section	Pre-class (Appendix A) 46 questions total	Post-class (Appendix B) 37 questions total	Follow-up (Appendix C) 48 questions total
**Actions measured at pre-class, intention measured at post-class, actions measured at follow-up**			
Practice characteristics	Systematic review participation (15, 16)	Intention to participate in systematic reviews (1, 2)	Systematic review participation since workshop (17, 18)
Practice characteristics	Awareness and use of practices from systematic review standards (17, 18, 23, 27 28)	Intention to learn about and use standards (7–16)	Reading, use of systematic review standards since workshop (19, 20, 25, 33, 34)
Practice characteristics	Asked for, received authorship (19, 20)	Intention to seek authorship (17, 18)	Asked for, received authorship (21, 22)
Practice characteristics	Provision of systematic review consultations to patrons (21)	Intention to provide patron consultations on systematic reviews (3, 4)	Provision of systematic review consultations to patrons since workshop (23)
Practice characteristics	Respondent and colleague systematic review education participation (24, 25, 26)	Intention to participate in future systematic review education (21, 22)	Respondent and colleague systematic review education participation since workshop (28, 29, 30)
Practice characteristics		Intention to use and share workshop materials (19, 20, 23, 24)	Use, sharing of workshop materials since workshop (26, 27, 31, 32)
**Same measures used at all time points**			
Practice characteristics	Confidence in systematic review skills (22, 29)	Confidence in systematic review skills (5, 6, 25, 26)	Confidence in systematic review skills (24, 35)
Knowledge questions	Knowledge (30–40)	Knowledge (27–37)	Knowledge (36–46)

Numbers in parentheses refer to item numbers in the survey instruments.

JMLA Journal of the Medical Library Association

Supplemental content to
J Med Libr Assoc. Jan;108(1):dx.doi.org/10.5195/jmla.2020.492
www.jmla.mlanet.org
© Folb, Klem, Youk, Dahm, He, Ketchum, Wessel, Hartman 2020

Table 2 Overall and individual survey response rates

**Overall response rate, pre-class to follow-up**							
	**Total invited pre-class**	**Completed follow-up**	**Follow-up incomplete but used**	**Opted out after completing pre class**	**Nonresponse for any survey**	**All responses used all three surveys**	**Overall response rate**
Pre-class to follow-up	160	99	4	6	51	103	64%
**Per survey response rate**							
**Survey**	**Invited**	**Completed**	**Incomplete, used**	**Incomplete, discarded**	**Nonresponse**	**Total responses used**	**Survey response rate**
Pre-class	160*	137	3	1§	19	140	88%
Post-class	136†	121	2	0	13	123	88%
Follow-up	122‡	99	4	0	19	103	84%

* All class attendees from July 2012–April 2014 invited.
† Nonrespondents to pre-class survey and respondents who requested to opt out of future surveys were not invited for post-class survey.
‡ Non respondents to post-class survey and respondents who requested to opt out of future surveys were not invited to follow-up survey.
§ Answered only demographic questions.

SC
Supplemental content to
J Med Libr Assoc. Jan;108(1):dx.doi.org/10.5195/jmla.2020.492
www.jmla.mlanet.org
© Folb, Klem, Youk, Dahm, He, Ketchum, Wessel, Hartman 2020

Table 3. Librarian characteristics, pre-class survey

Librarian characteristics	n	(%)
Job type (n=137)		
User services	108	(79%)
Management	12	(9%)
Other	17	(12%)
Years as a librarian (n=137)		
0–10	80	(58%)
11–20	29	(21%)
21–30	18	(13%)
31–40	10	(7%)
Degrees earned		
Master's of library science (MLS) (n=140)		
Yes	131	(94%)
No	9	(6%)
MLS plus subject masters (n=140)		
Yes	33	(24%)
No	98	(70%)
Missing	9	(6%)
MLS plus professional degree (n=140)		
Yes	5	(4%)
No	126	(90%)
Missing	9	(6%)

Table 4. Workplace characteristics, pre-class survey

Workplace characteristics	n	(%)
Type of organization (n=140)		
Health sciences library	94	(67%)
Academic library	15	(11%)
Hospital library	15	(11%)
Government library	9	(6%)
Other	7	(5%)
Librarians in workplace doing searches (n=140)		
1–5	75	(54%)
6–10	57	(41%)
11–15	8	(6%)
Library has a formal systematic review service? (n=140)		
Yes	38	(27%)
No, but plan to implement	44	(31%)
No, and no plans to implement	47	(34%)
Missing	11	(8%)

JMLA Journal of the Medical Library Association

Supplemental content to
J Med Libr Assoc. Jan;108(1):dx.doi.org/10.5195/jmla.2020.492
www.jmla.mlanet.org
© Folb, Klem, Youk, Dahm, He, Ketchum, Wessel, Hartman 2020

Table 5. Behavioral changes from pre-class to follow-up

	Pre-class (n varies)			Follow-up (n varies)				
	n	Positive responses	(%)	n	Positive responses	(%)	Z-test statistic	*p*-value
Did systematic review searching*	140	80	(58%)	103	65	(63%)	Z=–0.87	*p*=0.384
Sought peer review of own search*	80	29	(36%)	65	31	(48%)	Z=–1.39	*p*=0.164
Searched grey literature*	79	39	(49%)	64	34	(53%)	Z=–0.45	*p*=0.655
Asked for authorship*	80	27	(34%)	65	37	(57%)	Z=–2.79	*p*=0.005
Did peer review of another's search*	139	13	(9%)	103	17	(17%)	Z=–1.66	*p*=0.098
Read Institute of Medicine report*	135	39	(29%)	102	69	(68%)	Z=–5.93	*p*<0.0001
			Means			Means	Paired *t*-test statistic	*p*-value
Used guidelines (PRISMA)†	119		2.38	84		1.64	*t*=5.16	*p*<0.0001

* Test of proportion (Z-test) used.
† Paired *t*-test used. Likert scale data, response options: strongly agree=1, agree=2, neutral-3, disagree=4, strongly disagree=5.

Table 6. Reported and intended systematic review educational activities, pre-class, post-class, and follow-up

	Pre-class (n=140)		Post-class intention (n=123)		Follow-up (n=103)		Z-test statistic, pre-class to follow-up*	*p*-value, pre- class to follow-up
	n	(%)	n	(%)	n	(%)		
Attend systematic review trainings	52	(37%)	81	(66%)	30	(29%)	Z=1.34	*p*=0.178
Take curricular class	23	(16%)	—	—	10	(10%)	Z=1.51	*p*=0.131
Library staff in house education	86	(61%)	—	—	44	(43%)	Z=2.89	*p*=0.004
Use HSLS class materials	—	—	123	(100%)	96	(93%)	—	
Share HSLS class materials with colleagues	—	—	116	(94%)	86	(83%)	—	

Test of proportion (Z-test) used.

JMLA Journal of the Medical Library Association

SC
Supplemental content to
J Med Libr Assoc. Jan;108(1):dx.doi.org/10.5195/jmla.2020.492
www.jmla.mlanet.org
© Folb, Klem, Youk, Dahm, He, Ketchum, Wessel, Hartman 2020

Table 7. Staff systematic review education activities in the workplace, pre-class and follow-up

Educational activity	Pre-class (n=86)		Follow-up (n=44)	
	n	(%)	n	(%)
Attended local workshops	44	(51%)	12	(27%)
One on one mentoring	34	(40%)	22	(50%)
Journal club	21	(24%)	10	(23%)
Other group study activity	12	(14%)	5	(11%)
Invited speakers	11	(13%)	5	(11%)
HSLS workshop*	5	(6%)	2	(5%)
Other workshops*	3	(3%)	1	(2%)
Webinars*	3	(3%)	1	(2%)
Information sharing*	2	(2%)	3	(7%)
Books*	1	(1%)	0	–
Learn by doing*	1	(1%)	0	–
Audited classes*	1	(1%)	0	–
Library systematic review service activities*	0	–	1	(2%)
Systematic review email discussion list participation*	0	–	1	(2%)

Only respondents who indicated one or more activities are included in this table. Multiple answers possible.
* Indicates write in response submitted under "other" category.

Supplemental content to
J Med Libr Assoc. Jan;108(1):dx.doi.org/10.5195/jmla.2020.492
www.jmla.mlanet.org
© Folb, Klem, Youk, Dahm, He, Ketchum, Wessel, Hartman 2020

Table 8 Pre-class existence of formal systematic review service, plans for future service, and changes at follow-up

	Yes		No		Missing*			
**Pre-class SR services**	**n**	**(%)**	**n**	**%**	**n**	**(%)**		
Existing SR service? (n=129)*	38	(29%)	91	(71%)	11	(9%)		
If no, is one planned? (n=91)	44	(48%)	47	(52%)	–	–		
	**Yes**		**No**		**Don't know**		**Not applicable, already had one**	
**Follow-up SR service changes**	**n**	**(%)**	**n**	**(%)**	**n**	**(%)**	**n**	**(%)**
Were changes made to SR services? (n=103)	32	(31%)	66	(64%)	5	(5%)	–	–
If yes, was a formal SR service added? (n=32)	20	(63%)	9	(28%)	–	–	3	(9%)

* Missing all from one cohort due to error in survey question skip set up.
